# Ventilatory abnormalities in patients with cystic fibrosis undergoing the submaximal treadmill exercise test

**DOI:** 10.1186/s12890-015-0056-5

**Published:** 2015-05-19

**Authors:** Paloma Lopes Francisco Parazzi, Fernando Augusto de Lima Marson, Maria Angela Gonçalves de Oliveira Ribeiro, Celize Cruz Bresciani de Almeida, Luiz Cláudio Martins, Ilma Aparecida Paschoal, Adyleia Aparecida Dalbo Contrera Toro, Camila Isabel Santos Schivinski, Jose Dirceu Ribeiro

**Affiliations:** Department of Pediatrics, State University of Campinas, Tessália Vieira de Camargo 126, Cidade Universitária “Zeferino Vaz”, 13083-887 Campinas, SP Brazil; Department of Medical Genetics, State University of Campinas, Tessália Vieira de Camargo 126, Cidade Universitária “Zeferino Vaz”, 13083-887 Campinas, SP Brazil; Clinical Hospital, State University of Campinas, Tessália Vieira de Camargo 126, Cidade Universitária “Zeferino Vaz”, 13083-887 Campinas, SP Brazil; Department of Clinical Medics of the Faculty of Medical Sciences, State University of Campinas, Tessália Vieira de Camargo 126, Cidade Universitária “Zeferino Vaz”, 13083-887 Campinas, SP Brazil; State University of Santa Catarina, Center of Physical Education and Sports, Coqueiros, 88080-350 Florianópolis, SC Brazil

**Keywords:** Cystic fibrosis, 6-minute walk test, Spirometry, Volumetric capnography

## Abstract

**Background:**

Exercise has been studied as a prognostic marker for patients with cystic fibrosis (CF), as well as a tool for improving their quality of life and analyzing lung disease. In this context, the aim of the present study was to evaluate and compare variables of lung functioning. Our data included: (i) volumetric capnography (VCAP) parameters: expiratory minute volume (VE), volume of exhaled carbon dioxide (VCO_2_), VE/VCO_2_, ratio of dead space to tidal volume (VD/VT), and end-tidal carbon dioxide (PetCO_2_); (ii) spirometry parameters: forced vital capacity (FVC), percent forced expiratory volume in the first second of the FVC (FEV_1_%), and FEV_1_/FVC%; and (iii) cardiorespiratory parameters: heart rate (HR), respiratory rate, oxygen saturation (SpO_2_), and Borg scale rating at rest and during exercise. The subjects comprised children, adolescents, and young adults aged 6–25 years with CF (CF group [CFG]) and without CF (control group [CG]).

**Methods:**

This was a clinical, prospective, controlled study involving 128 male and female patients (64 with CF) of a university hospital. All patients underwent treadmill exercise tests and provided informed consent after study approval by the institutional ethics committee. Linear regression, Kruskal–Wallis test, and Mann–Whitney test were performed to compare the CFG and CG. The α value was set at 0.05.

**Results:**

Patients in the CFG showed significantly different VCAP values and spirometry variables throughout the exercise test. Before, during, and after exercise, several variables were different between the two groups; statistically significant differences were seen in the spirometry parameters, SpO_2_, HR, VCO_2_, VE/VCO_2_, PetCO_2_, and Borg scale rating. VCAP variables changed at each time point analyzed during the exercise test in both groups.

**Conclusion:**

VCAP can be used to analyze ventilatory parameters during exercise. All cardiorespiratory, spirometry, and VCAP variables differed between patients in the CFG and CG before, during, and after exercise.

**Electronic supplementary material:**

The online version of this article (doi:10.1186/s12890-015-0056-5) contains supplementary material, which is available to authorized users.

## Background

The role of exercise as a prognostic indicator and/or therapeutic instrument is of interest in the research of several diseases, particularly respiratory diseases. Over the past three decades, exercise has become well established as an important component in the management of cystic fibrosis (CF) [1].

From birth, patients with CF undergo progressive deterioration of lung structures [1-5]. This deterioration of anatomical structures causes functional changes directly related to respiratory function. Changes include decreases in the peripheral oxygen saturation (SpO_2_), forced expiratory volume in the first second of the forced vital capacity (FEV_1_), ratio of dead space to tidal volume (VD/VT) and exercise performance, increases in the respiratory rate (RR) and heart rate (HR), and changes in blood gas parameters.

The benefits of exercise in patients with CF include improved aerobic conditioning, decreased progression of lung disease, and enhanced bronchial hygiene through an increased ciliary beat frequency, activation of immune cells, reduced susceptibility to viral infections, and increased anti-inflammatory activity. Thus, patients experience a better quality of life [1,6-8].

The exercise stress test has been increasingly used to assess the level of exercise intolerance in patients with heart and lung diseases. This test is considered the gold standard for the study of exercise limitations and their causes [9,10].

Volumetric capnography (VCAP) analyzes the pattern of elimination of carbon dioxide (CO_2_) in the expired air volume and can reveal a link between early structural damage and functional lung abnormalities in patients with CF. Previous studies have evaluated VCAP as a tool for assessing the degree of regional heterogeneity of lung gas exchange [11-13]. Some studies have found a relationship between VCAP parameters and pulmonary involvement indices derived from VCAP [14-16]. The curve of expired CO_2_ over time, known as a capnograph, can show temporal changes in obstructive lung disease. Besides the volume of exhaled CO_2_ (VCO_2_), VCAP measures the expiratory minute volume (VE), HR, RR, SpO_2_, end-tidal CO_2_ (PetCO_2_), and VD/VT ratio, which in normal subjects decreases during exercise and increases when imbalance occurs [17,18].

Ribeiro et al. [19] recently compared VCAP and spirometry in 64 children and adolescents with CF and 94 healthy subjects. Patients with CF showed a higher slope3/VT than did healthy subjects. VCAP also showed a difference in the slope3/VT in patients with CF who had normal spirometry results, indicating an early periphery airway dysfunction. An increased slope3/VT reflects inhomogeneity of ventilation in the distal airways, suggesting the possibility of chronic structural dysfunction as well as reversible acute dysfunction that may be observed, for example, in a bronchoprovocation test. This index may be a useful tool in the evaluation and study of small airway dysfunction in children and adolescents with pulmonary disease.

The positive impact of regular physical activity and the need for information about its effects justify the growing interest in studies on regular exercise as a part of treatment for patients with CF and as a tool to assess pulmonary deterioration. This illustrates the relevance of correlating the exercise stress test results with prognostic indices obtained by VCAP. Moreover, comparison of the results obtained from these tests between patients with CF and healthy individuals demonstrates the importance of physical and pulmonary rehabilitation and justifies the incorporation of exercise in the routine treatment of patients with CF.

In this context, the aim of this study was to evaluate VCAP parameters (VE, VCO_2_, VD/VT, VE/VCO_2_, and PetCO_2_) and cardiorespiratory parameters (HR, RR, and SpO_2_) at rest and during the 6-minute walk test between patients with CF and healthy subjects and to compare these values with spirometry data and clinical markers.

## Methods

This clinical, prospective, controlled study involved male and female patients with CF (CF group [CFG]) from a university hospital and healthy volunteers (control group [CG]).

All patients with CF who agreed to participate were included. All patients aged >18 years and the caregivers of patients aged <18 years provided written informed consent. The inclusion criteria were an age of 6–25 years and the presence of CF diagnosed according to the criteria of the international consensus [20,21] by a sweat test with a chloride level of >60 mEq/L and positive genetic screening for the CF transmembrane regulator (*CFTR*) mutation [20,22], when possible.

Patients were not in a state of pulmonary exacerbation as verified by the application of two clinical scores: the Cystic Fibrosis Foundation Clinical Score and the Cystic Fibrosis Clinical Score [23,24]. The CG included children, adolescents, and young adults aged 6–25 years who were randomly selected among students from public and private schools in the same university district. All participants answered a questionnaire, and no subjects had other acute or chronic diseases.

The exercise stress test was performed on a treadmill (Pro Cl 5004; Caloi-Electronic, Wallbach, Switzerland). The velocity was increased according to each individual’s tolerance level until a submaximal test level was reached; at no time during the test did any individual exceed 75% of their maximum HR, which was calculated by 220 − the age of the individual [25].

Before starting the exercise test, all patients in the CFG and CG underwent spirometry according to the guidelines of the European Respiratory Society and American Thoracic Society. The test started by measuring cardiorespiratory variables at rest for 3 minutes; this was followed by measurements during exercise testing on the treadmill for 6 minutes and immediately at the end of the test for a further 3 minutes. Patients in both the CG and CFG followed the same test sequence. The same trained professional performed all examinations.

Initial spirometry was performed using a model CPFS/D spirometer (MedGraphics, Saint Paul, MN, USA) with PF BREEZE Software, Version 3.8B for Windows 95/98/NT (MedGraphics).

For VCAP, the CO2SMO Plus DX 8100 monitor (Novametrix Medical Systems, Wallingford, CT, USA) was used. This is a noninvasive monitor with a capnograph, pulse oximeter, and pneumotachograph. The monitor is connected to CO_2_ flux and pulse oximetry sensors. The CO_2_ and flow sensors were used in all subjects and discarded after each use according to the specifications in the manual. These flow and CO_2_ sensors are combined at their distal portions and coupled to a nozzle. The pediatric/adult (#6719) model of the flow sensor was used. Capnography and pneumotachography measures were obtained in real time by analyzing the gases breathed. The CO2SMO Plus was connected to a computer equipped with software to record the flow, volume, pressure, pressure–volume, flow–volume, and capnography measurements and curves.

The sensor monitor was connected to a mouthpiece, and a nose clip was used to prevent air escaping. Participants were seated in a quiet room and breathed for 1 minute to adapt to the equipment. The monitoring was then started with Analysis Plus® software. Participants maintained normal, relaxed breathing for 4 minutes, and the variables were recorded and stored on the computer. At the end of the data collection, an offline sequence of breaths was selected to determine the coefficient of variation for the lowest expired VT of 25% of the average VT; for PetCO_2_, measured in mmHg, a lower coefficient of variation of 10% was permitted. Breaths that presented a value of zero for the slope3 variable were excluded. The results were obtained through the average of the parameters collected during the 4-minute monitoring.

The analysis of expiratory gases was performed throughout the test, with each patient using his or her own mouthpiece and nose clip from beginning to end. All subjects received prior clarification regarding the test to be performed.

### Statistical analysis

For patients in both the CG and CFG, statistical analysis was performed at five different time points during the exercise test: (1) baseline, (2) 1–2 minutes of activity, (3) 3–4 minutes of activity, (4) 5–6 minutes of activity, and (5) immediately postexercise. The data were processed using Statistical Package for Social Sciences, Version 21.0 software (SPSS Inc., Chicago, IL, USA). For numerical data, the mean, confidence interval, standard deviation, median, and minimum and maximum values were used. For categorical data, the absolute number and percentage were used.

Comparisons between the CG and CFG were performed with the Mann–Whitney test for variables with a numeric distribution, and calculations of differences in categorical variables between the CG and CFG were performed with the χ^2^ test. Correlations between these time points in each group were determined using the Friedman test (non-parametric, repeated measures), and in cases with a positive p-value, the Wilcoxon signed-rank test was performed. Linear regression was performed to compare the FEV_1_% and the VCAP parameter VCO_2_ at all time points analyzed. Linear regression was also performed to evaluate VCO_2_ in relation to the body mass index (BMI) of all subjects.

For all analyses, we used a value of α = 0.05 with correction for multiple testing by the Bonferroni method (α = 0.05 / 5 = 0.01).

The research ethics committee of the State University of Campinas (#1182/2009) approved this study.

## Results

In total, 64 patients with CF and 64 healthy volunteers of both sexes with no statistical differences in age or sex were included in the study (p > 0.05). The complete genotypes of mutations in the *CFTR* gene are shown in Additional file 1: Table S1. A description of each identified mutation in the *CFTR* gene is presented in Additional file 2: Table S2.

Weight (kg), height (m^2^), and BMI (kg/m^2^) were significantly lower in the CFG (34.80 ± 13.30, 1.42 ± 0.19, and 16.72 ± 3.15, respectively) than in the CG (46.39 ± 14.44, 1.52 ± 0.13, and 19.56 ± 3.33, respectively) (p ≤ 0.001) (Table 1).

**Table 1:** **Distribution of patients with cystic fibrosis and healthy subjects according to clinical laboratory markers**

**Marker**	**Patients with cystic fibrosis**	**Healthy subjects**	**p-value**
Sex (male)	32 (51.61)	30 (48.39)	0.860
Sex (female)	32 (48.50)	34 (51.50)	
Age (years)	12.34 (11.48–13.21) ± 3.47	13.51 (12.34–14.68) ± 4.64	0.490
	12.00 (6.00–23.00)	11.00 (9.00–25.00)	
Weight (kg)	34.80 (31.47–38.12) ± 13.30	46.39 (42.75–50.02) ± 14.44	≤0.001
	33.75 (12.70–74.20)	44 (25.80–87.90)	
Height (m)	1.42 (1.37–1.46) ± 0.19	1.52 (1.49–1.56) ± 0.132	0.003
	1.45 (1.00–1.74)	1.58 (1.20–1.87)	
BMI	16.72 (15.93–17.50) ± 3.15	19.56 (18.72–20.40) ± 3.33	≤0.001
	15.87 (11.30–28.63)	19.01 (13.46–27.13)	
Shwachman–Kulczycki score	77.93 (76.20–79.67) ± 6.94	–	–
	80.00 (60.00–90.00)	–	
FVC%	78.61 (73.44–83.78) ± 20.68	95.78 (92.89–98.67) ± 11.56	≤0.001
	80.50 (32.00–121.00)	93.00 (69.00–130.00)	
FEV_1_%	68.98 (63.38–74.59) ± 22.45	91.94 (87.70–96.17) ± 16.96	≤0.001
	65.50 (19.00–117.00)	90.00 (41.00–125.00)	
FEV_1_/FVC	85.66 (82.77–88.54) ± 11.56 87.50 (52.00–107.00)	98.23 (95.99–100.47) ± 8.95 100.00 (74.00–125.00)	≤0.001
Borg rating (initial)			<0.001
0	48 (75.0%)	64 (100.0%)	
1	12 (19.0%)	0 (0.0%)	
2	3 (5.0%)	0 (0.0%)	
3	1 (1.0%)	0 (0.0%)	
Borg rating (end)			
0	33 (52.0%)	56 (88.0%)	
1	6 (9.0%)	1 (1.0%)	
2	14 (22.0%)	4 (6.3%)	
3	6 (9.0%)	0 (0.0%)	
4	2 (3.0%)	0 (0.0%)	≤0.005
5	1 (1.0%)	0 (0.0%)	
6	1 (1.0%)	2 (3.0%)	
7	0 (0.0%)	1 (1.0%)	
10	1 (1.0%)	0 (0.0%)	

FVC, forced vital capacity; FEV_1_, forced expiratory volume in the first second of the FVC; BMI, body mass index. For sex and the Borg scale rating, the data are presented as *n* (%). For variables with numeric distribution, the data are presented as mean (confidence interval) ± standard deviation and median (minimum–maximum). Differences between patients with CF and healthy subjects were calculated by the χ^2^ test for categorical variables and by the Mann–Whitney test for numerical data not normally distributed (α = 0.05).

The Shwachman–Kulczycki score in the CFG ranged from 60 to 90 (77.93 ± 6.94) (Table 1).

In both groups, assessment of dyspnea using the modified Borg scale (Table 1) showed statistically significant differences before (p ≤ 0.001) and after testing (p ≤ 0.005).

Spirometric values at rest for both groups are shown in Table 1. Patients in the CFG had a significantly lower FVC, FEV_1_, and FEV_1_/FVC: (78.61 ± 20.68, 68.98 ± 22.45, and 85.66 ± 11.56, respectively) than did patients in the CG (95.78 ± 11.56, 91.94 ± 16.96, and 98.23 ± 8.95, respectively) (p ≤ 0.001) (Table 1).

The complete list of VCAP values according to the time of analysis between the CG and CFG is shown in Additional file 3: Table S3.

**Figure 1:**
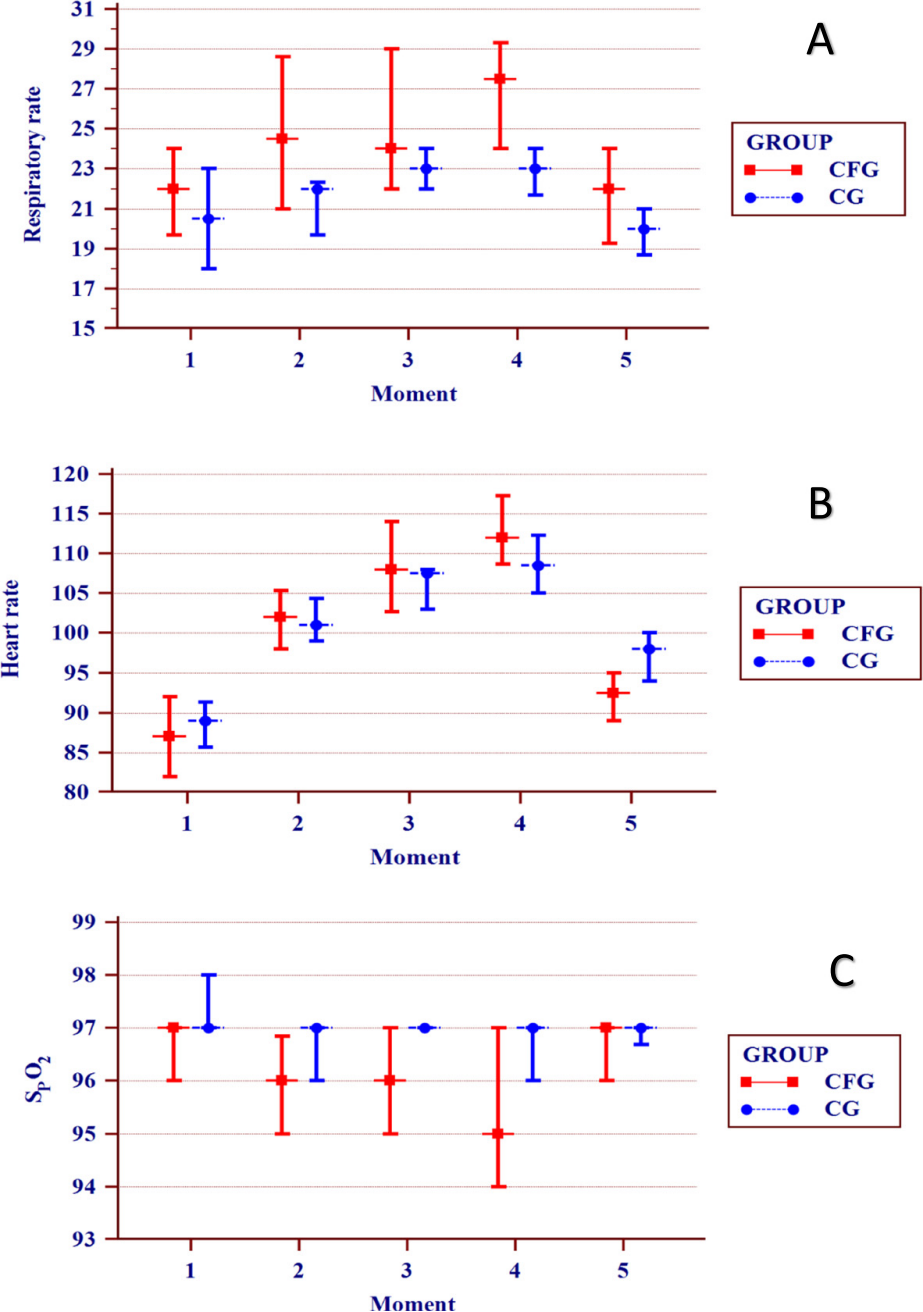
Comparison of cardiorespiratory variables between the group of patients with cystic fibrosis (CFG) (n=64) and the control group (CG) (n=64). Data are presented as the confidence interval of the median. Time points analyzed: (1) baseline (rest), (2) 1–2 min of activity, (3) 3–4 min of activity, (4) 5–6 min of activity, and (5) postexercise. α=0.01 by Bonferroni correction. The Mann–Whitney test was used to compare the CFG and CG, and correlations between the time points in each group were determined using the Friedman test (non-parametric, repeated measures); in cases with a positive p-value, the Wilcoxon signed-rank test was performed. **(A)** Respiratory rate. There was no difference between the CG and CFG for the time points analyzed. In the cluster analysis for the CFG, time point 1 differed from 2 to 4; time point 2 differed from 1; time points 3 and 4 differed from 1 and 5; and time point 5 differed from 3 and 4 (p≤0.001). In the cluster analysis for the CG, time points 3 and 4 differed from 5, and time point 5 differed from 3 and 4 (p≤0.001). **(B)** Heart rate. There was no difference between the CG and CFG for the time points analyzed. In the cluster analysis for the CFG, time point 1 differed from 2 to 4; time point 2 differed from 1, 4, and 5; time point 3 differed from 1 and 5; time point 4 differed from 1, 2, and 5; and time point 5 differed from 2 to 4 (p≤0.001). In the cluster analysis for the CG, time point 1 differed from 2 to 5; time point 2 differed from 1, 3, 4, and 5; time points 3 and 4 differed from 1, 2, and 5; and time point 5 differed from 1 to 4 (p≤0.001). **(C)** Transcutaneous oxygen saturation. There was no difference between the CG and CFG for time points 4 and 5. For the other time points, the CG exhibited higher values (time points 1 and 2, p≤0.001; time point 3, p=0.002). In the group analysis for the CFG, time point 1 differed from 2 and 4; time point 2 differed from 1 and 5; time point 3 differed from 5; time point 4 differed from 1 and 5; and time point 5 differed from 2 to 4 (p≤0.001). In the CG, time point 1 differed from 2 to 5 (p≤0.001).

The RR was higher in the CFG than in the CG at all times analyzed, but the differences were not statistically significant (Figure 1).

The HR was not different between the two groups at any time points analyzed (p > 0.05). The SpO_2_ was lower in the CFG (93.98 ± 7.22) than in the CG (95.90 ± 5.32), but the difference between time points 4 and 5 was not statistically significant (p ≤ 0.001) (Figure 1B, 1C).

**Figure 2:**
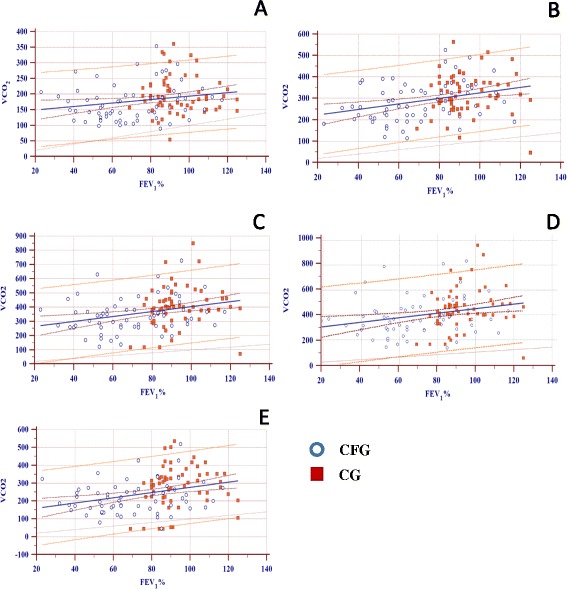
Distribution points for linear regression of FEV_1_% and VCO_2_. **A**. Time point 1 - [Combined: y = 0.599 x + 134.183 (p = 0.011); R^2^ = 0.0495], [CF: y = 0.547 x + 132.642 (p = 0.091); R^2^ = 0.045], [Control: y = 197.146 – 0.0195 x (p = 0.973); R^2^ = 0.000018]. **B**. Time point 2 - [Combined: y = 1.290 x + 196.511 (p = 0.0006); R^2^ = 0.09095], [CF: y = 1.408 x + 184.347 (p = 0.005); R^2^ = 0.1198], [Control: y = 291.749 + 0.313 x (p = 0.736); R^2^ = 0.001876]. **C**. Time point 3 - [Combined: y = 1.695 x + 234.349 (p = 0.001); R^2^ = 0.079], [CF: y = 1.256 x + 254.893 (p = 0.063); R^2^ = 0.05544], [Control: y = 254.560 + 1.584 x (p = 0.253); R^2^ = 0.02173]. **D**. Time point 4 - [Combined: y = 1.805 x + 269.757 (p = 0.005); R^2^ = 0.06034], [CF: y = 1.219 x + 300.252 (p = 0.164); R^2^ = 0.03098], [Control: y = 2.114 x + 250.909 (p = 0.189); R^2^ = 0.02815]. **E**. Time point 5 - [Combined: y = 237.724 + 1.548 x (p = 0.002); R^2^ = 0.0740], [CF: y = 251.939 + 1.248 x (p = 0.058); R^2^ = 0.05680], [Control: y = 249.636 + 1.492 x (p = 0.244); R^2^ = 0.02256].

There was an association between the distribution of FEV_1_(%) and VCO_2_ at all times during the 6-minute walk test (Figure 2).

**Figure 3:**
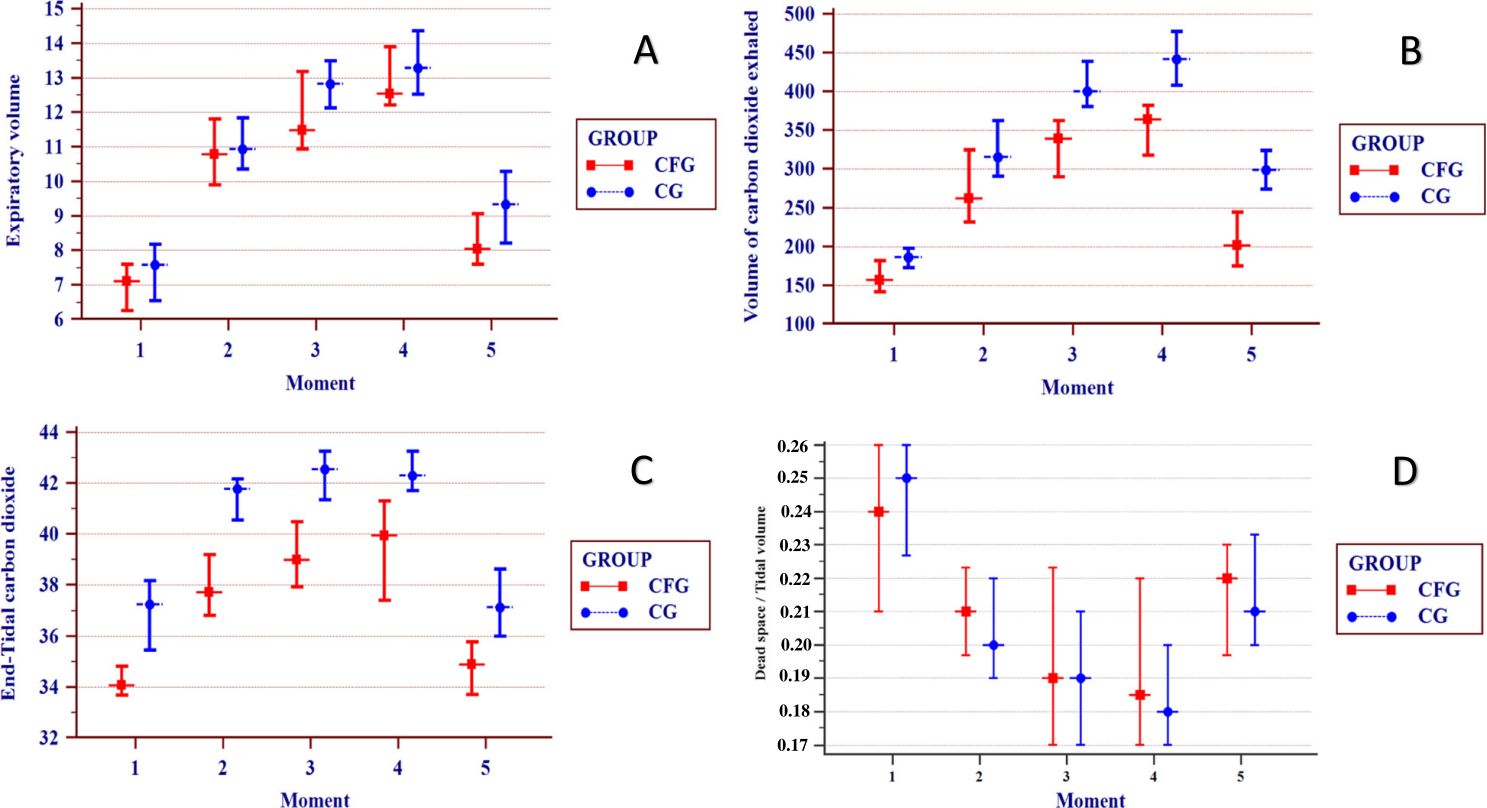
Variables from volumetric capnography between the group of patients with cystic fibrosis (CFG) (n=64) and the control group (CG) (n=64). Data are presented as the confidence interval of the median. Time points analyzed: (1) baseline (rest), (2) 1–2 min of activity, (3) 3–4 min of activity, (4) 5–6 min of activity, and (5) postexercise. α=0.01 by Bonferroni correction. The Mann–Whitney test (data not normally distributed) was used to compare the CFG and CG, and correlations between the time points in each group were determined using the Friedman test (non-parametric, repeated measures); in cases with a positive p-value, the Wilcoxon signed-rank test was performed. **(A)** Expired minute volume (VE). There was no difference between the CF and CFG for the different time points analyzed. In the cluster analysis, for the CFG, time point 1 differed from 2 to 5; time point 2 differed from 1, 4, and 5; time point 3 differed from 1 and 5; time point 4 differed from 1, 2, and 5; and time point 5 differed from 1 to 4 (p≤0.001). For the CG, time point 1 differed from 2 to 5; time point 2 differed from 1, 3, 4, and 5; time point 3 differed from 1, 2, and 5; and time point 5 differed from 1 to 4 (p≤0.001). **(B)** VCO_2_. Higher values were observed in the CG than CFG (time point: (1) p=0.003; (2) p=0.005; (3) p=0.001; (4) p=0.002; (5) p≤0.001). In the cluster analysis, for the CFG, time point 1 differed from 2 to 5; time point 2 differed from 1, 3, 4, and 5; time point 3 differed from 1, 2, and 5; and time point 5 differed from 1 to 4 (p≤0.001). For the CG, time point 1 differed from 2 to 5; time point 2 differed from 1, 3, and 4; time points 3 and 4 differed from 1, 2, and 5; and time point 5 differs from 1, 3, and 4 (p≤0.001). **(C)** PetCO_2_. Higher values were observed in the CG than CFG (time point 1, p = 0.002; other time points, p≤0.001). In the cluster analysis for the CFG, time point 1 differed from 2 to 4; time points 2 to 4 differed from 1 and 5; and time point 5 differed from 2 to 4 (p≤0.001). For the CG, time point 1 differed from 2 to 4; time point 2 differed from 1, 4, and 5; time point 3 differed from 1 and 5; time point 4 differed from 1, 2, and 5; and time point 5 differed from 2 to 4 (p≤0.001). **(D)** VD/VT. There was no difference between the CG and CFG for the different time points analyzed. In the cluster analysis for the CFG, time point 1 differed from 2 to 4; time point 2 differed from 1; time points 3 and 4 differed from 1 and 5; and time point 5 differed from 3 and 4 (p≤0.001). For the CG, time point 1 differed from 2 to 5; time point 2 differed from 1; time points 3 and 4 differed from 1 and 5; and time point 5 differed from 1, 3, and 4 (p≤0.001).

The VE/VCO_2_ increased throughout the test and decreased at the end in both groups, but without statistical significance (Figure 3A).

The Exhaled VCO_2_ increased in both groups and decreased at the end of the test, with statistical differences at all time points analyzed; higher values were observed in the CG (391.16 ± 128.98) than in the CFG (338.03 ± 117.55) (p ≤ 0.001) (Figure 3B).

The PetCO_2_ increased over time; higher values were observed in the CG (42.00 ± 3.82) than in the CFG (38.41 ± 5.73) (p ≤ 0.001) (Figure 3C).

The VD/VT ratio decreased over time and then returned to baseline; higher values were observed in the CFG, but without statistical significance (p > 0.05) (Figure 3D).

**Figure 4:**
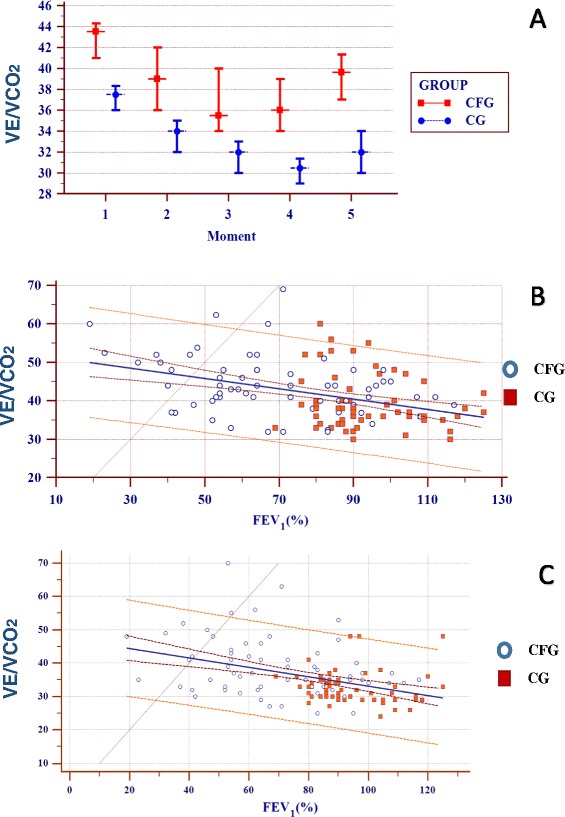
VE/VCO_**2**_ by confidence intervals in relation to different time points and linear regression for time points 1 and 5 with respect to FEV_1_%. **(A)** Confidence interval of VE/VCO_2_ for the cystic fibrosis group (CFG) (n=64) and control group (CG) (n=64). Data are presented as the confidence interval of the median. Time points analyzed: (1) baseline (rest), (2) 1–2 min of activity, (3) 3–4 min of activity, (4) 5–6 min of activity, and (5) immediately postexercise. α=0.01 by Bonferroni correction. The Mann–Whitney test was used to compare the CFG and CG, and correlations between the time points in each group were determined using the Friedman test (non-parametric, repeated measures); in cases with a positive p-value, the Wilcoxon signed-rank test was performed. Higher values were observed in the CG at all time points (p≤0.001). In the cluster analysis for the CFG, time point 1 differed from 2 to 5; time point 2 differed from 1 and 4; time point 3 differed from 1 and 5; time point 4 differed from 1, 2, and 5; and time point 5 differed from 1, 3, and 4 (p≤0.001). For the CG, time point 1 differed from 2 to 5; time point 2 differed from 1, 3, and 4; time point 3 differed from 1, 2, and 4; time point 4 differed from 1, 2, 3, and 5; and time point 5 differed from 1 and 4 (p≤0.001). **(B)** Linear regression of the VE/VCO_2_ and FEV_1_%. Time point 1 - [(Combined: y= 52.5615 – 0.1343x) (p≤0.0001); R^2^=0.01522; (CFG: y= 51.8380 – 0.1139x) (p=0.0056); R^2^=0.1122; (CG: y = 48.0635 – 0.0935x) (p=0.1756); R^2^=0.02986]. **(C)** Linear regression of the EV/VCO_2_ and FEV_1_%. Moment 5 - [(Combined: y= 47.2563 – 0.1411x) (p≤0.001); R^2^=0.1622; (CFG: y= 46.6691 – 0.1139x) (p=0.024); R^2^=0.08373; (CG: y= 35.5437 – 0.02961x) (p=0.5310); R^2^=0.006467].

The VE/VCO_2_ ratio decreased during the test and increased at the end. A statistically significant difference was observed at all time points analyzed, and patients in the CFG had higher ratios at the end of the test (38.81 ± 8.83) than did patients in the CG (32.72 ± 4.74) (p ≤ 0.001) (Figure 4A). The distribution of FEV_1_(%) showed an inverse association with the VE/VCO_2_ at time points 1 and 5 in both the CFG and CG (Figure 4B and C**)**.

Linear regression of VCO_2_ in relation to BMI is shown in Additional file 4: Figure S4

All children completed the test without exhibiting cardiorespiratory symptoms requiring cessation of the test.

## Discussion

Exercise has been studied as a tool with which to assess the improvement or worsening of cardiorespiratory function [26-29]. To the best of our knowledge, this is the first study to evaluate ventilatory efficiency through the use of VCAP before, during, and after exercise between patients with and without CF. Furthermore, it reinforces the positive effects of the association between exercise and therapy in these patients. Few studies have been conducted using VCAP for evaluation of CF [19,30,31].

Our results reinforce the importance of exercise as a useful tool in cardiorespiratory evaluation of patients with CF. All cardiorespiratory, spirometry, and VCAP markers differed between the CFG and CG before and after exercise.

It is well known that CF causes progressive deterioration in the ability to perform physical exercise. However, regardless of severity, both children and adults may develop increased ability to tolerate exercise and benefit from it in the long term [28].

Various authors have studied transverse or longitudinal models and the benefits of exercise on lung function in patients with severe CF [27,29]. However, our population of patients with CF had higher FEV_1_ values than those in other studies [26,27]. This reflects a population with minor deterioration in lung function. There are several potential explanations for this difference; for example, the patients in our center received medications at no cost and underwent care by multidisciplinary teams, the *CFTR* mutations belonged to different classes, early diagnosis was achieved, interdisciplinary monitoring was performed, and the patients included in our study were relatively young.

Various markers of the deterioration of lung function in patients with CF have been investigated and include clinical and tomographic scores, spirometry values, VCAP values, the lung clearance index, and others [19]. One confirmed paradigm is that high-resolution computed tomography detects changes in lung function earlier than does spirometry, which illustrates the need for new tools with which to investigate lung function. The use of VCAP has not been extensively studied in patients with CF. Hopefully, it will become the focus of new studies to enable more accurate diagnosis of early pulmonary disease [19,30,31]. Thus, in the present study, VCAP was analyzed as a practical, inexpensive, and easy-to-perform method that can give important information about ventilatory function.

Throughout the exercise test, we observed that the RR and HR were higher in the CFG than in the CG at all time points, and the HR was higher in the CG only immediately after exercise. This finding is in agreement with a recent study by Pereira et al. [8] in which 55 patients with CF and 185 healthy individuals were evaluated during the 6-minute walk test. During the test, patients with CF exhibited a lower SpO_2_ than did healthy individuals. The same result was observed by Holland et al. [32] in a study of 101 adults with moderate to severe CF performing the 3-minute step test. The authors reported that desaturation during the test was associated with long-term pulmonary deterioration and longer hospitalization periods in adults with CF. In a study by Pereira et al. [8], SpO_2_ remained stable during the 6-minute walk test.

In the present study, evaluation of dyspnea using the modified Borg scale showed that the highest dyspnea ratings were found in the CFG before and after the test; this is in agreement with the data reported by Pereira et al. [8].

With progressive deterioration of the lungs, the amount of VD increases, requiring changes in ventilation to maintain adequate alveolar ventilation during exercise. Thus, changes in lung function over time have been correlated with changes in exercise capacity. CO_2_ retention during the maximal exercise stress test also reportedly contributed to a faster rate of decline in FEV_1_% in a study of children with CF aged 11–15 years [26].

Throughout our analysis, patients in the CFG showed significantly greater retention of Exhaled VCO_2_ than did patients in the CG, who exhaled larger amounts of VCO_2_. This finding can be explained by dysfunction that causes difficultly in gas exchange, such as disruption of the lung architecture, destruction of parts of the capillary bed, increased DS, and secretion retention [33-35]. In the present study, we chose not to correct the VCAP values by the BMI because the lower value of BMI in the CFG was associated with the patients’ clinical disease status, was a common factor in the population tested, and represented the clinical picture at each time point analyzed. If such a correction were made, the obtained data would mask the true severity of the disease.

Assessment of the cardiorespiratory responses of healthy children during the maximal exercise test revealed that the responses were higher for HR, RR, VCO_2_, VD/VT, and PetCO_2_, while VT and PetCO_2_ showed a smaller increase in children than in adults. At peak exercise, larger differences were observed in HR and VD/VT in children than in adults, suggesting there is increased ventilation in the anatomical VD [36].

Leroy et al. [27] studied the impact of dyspnea and alveolar hypoventilation during exercise in 18 patients with CF through a maximal exercise test on a cycle. They found that during exercise, patients exhibited progressive increases in their VT, reaching a peak VT at 48% of FVC [27]. The VD/VT ratio also increased during exercise and represented 34% of the VT [27].

A main limitation of our study is the exclusion of a curve of normal VCAP values. Although our sample has statistical power for analysis, future studies with larger numbers of patients of the same age and *CFTR* mutation class should be performed.

## Conclusions

We conclude that VCAP can be used as a tool for analysis of ventilatory efficiency during exercise. All cardiorespiratory, spirometry, and VCAP variables differed between patients with CF and healthy subjects before and after exercise. These results indicate imbalanced ventilation/perfusion characterized by both hypoxemia and CO_2_ retention, which compromises exercise tolerance in patients with CF. Continued research of cardiorespiratory markers in transverse and longitudinal models at rest and after exercise is needed to better understand the deterioration of lung function in patients with CF.

## Additional files

Additional file 1: Table S1. Genotypes for the *CFTR* mutations of patients with cystic fibrosis enrolled in the present study (n = 64). Additional file 2: Table S2.
*CFTR* mutations found in individuals enrolled in the study. Gene and protein localization. Mutation classification and frequency from the present study are designated. Traditional and Human Genome Variation Society standard nomenclature for *CFTR* mutations are also indicated. Additional file 3: Table S3. Volumetric capnograph markers taking into account groups of patients with cystic fibrosis and healthy subjects. Time points analyzed by groups. Additional file 4: Figure S4. Linear regression of VCO_2_ and FEV_1_% by body mass index (BMI). **A**. Time point 1 - VCO_2_ by BMI - [Combined: y = 12.382 + 0.031 x (p ≤ 0.001)], [CF: y = 12.381 + 0.025 x (p = 0.001)], [Control: y = 13.810 + 0.029 x (p ≤ 0.001)]. **B**. Time point 3 - VCO_2_ by BMI - [Combined: y = 12.573 x + 0.015 (p ≤ 0.001)], [CF: y = 12.278 + 0.013 x (p < 0.001)], [Control: y = 14.244 + 0.013 x (p ≤ 0.001)]. **C**. Time point 5 - VCO_2_ by BMI - [Combined: y = 11.854 x + 0.017 (p ≤ 0.001)], [CF: y = 11.712 + 0.015 x (p ≤ 0.001)], [Control: y = 13.266 + 0.016 x (p ≤ 0.001)]. **D.** Time point 1 - FEV_1_% by BMI - [Combined: y = 13.566 + 0.056 x (p = 0.001)], [CF: y = 15.127 + 0.023 x (p = 0.196)], [Control: y = 14.919 + 0.049 x (p = 0.133)].

## Abbreviations

VCAP: Volumetric capnography
CFTR: Cystic fibrosis transmembrane regulator
CO_2_: Carbon dioxide
FVC: Forced vital capacity
CF: Cystic fibrosis
HR: Heart rate
RR: Respiratory rate
CG: Control group
CFG: Cystic fibrosis group
BMI: Body mass index
PetCO_2_: End-tidal carbon dioxide
SpO_2_: Oxygen saturation
VCO_2_: Volume of exhaled carbon dioxide
VD: Dead space
VE: Expiratory minute volume
FEV_1_: Forced expiratory volume in the first second of the forced vital capacity
VT: Tidal volume
VE/VCO_2_: Ventilatory efficiency index
