# Supplementary material for: Ventilatory abnormalities in patients with cystic fibrosis undergoing the submaximal treadmill exercise test
Source: BMC Pulm Med. 2015 May 19;15:63. doi: 10.1186/s12890-015-0056-5 (PMC4446830; doi:10.1186/s12890-015-0056-5)
Supplement: Additional file 1: Table S1. — Genotypes for the CFTR mutations of patients with cystic fibrosis enrolled in the present study (n = 64). [file 12890_2015_56_MOESM1_ESM.docx]

| **Supplement 1.** Genotypes for the *CFTR* mutations of patients with cystic fibrosis enrolled in the present study (n = 64) | | | |
| --- | --- | --- | --- |
| **Mutation** | **Patients (n)** | **%** | **Cumulative %** |
| F508del/F508del | 22 | 34.4 | 34.4 |
| F508del/G542X | 7 | 10.9 | 45.3 |
| F508del/1812-1G>A | 2 | 3.1 | 48.4 |
| F508del/c.1717-1G>A | 2 | 3.1 | 51.5 |
| F508del/N1303K | 2 | 3.1 | 54.6 |
| 3120+1G>A/R1066C | 2 | 3.1 | 57.7 |
| F508del/2183AA>G | 1 | 1.6 | 59.3 |
| F508del/R1066C | 1 | 1.6 | 60.9 |
| F508del/R1162X | 1 | 1.6 | 62.5 |
| F508del/R553X | 1 | 1.6 | 64.1 |
| 2183AA>G/2183AA>G | 1 | 1.6 | 65.7 |
| R1162X/R1162X | 1 | 1.6 | 67.3 |
| F508del/NMI | 8 | 12.5 | 79.8 |
| G542X/NMI | 1 | 1.6 | 81.4 |
| I507V/NMI | 1 | 1.6 | 83 |
| R1162X/NMI | 1 | 1.6 | 84.6 |
| NMI/NMI | 10 | 15.6 | 100 |

*CFTR* = cystic fibrosis transmembrane regulator gene, NMI = no mutation identified.
