# Supplementary material for: Ventilatory abnormalities in patients with cystic fibrosis undergoing the submaximal treadmill exercise test
Source: BMC Pulm Med. 2015 May 19;15:63. doi: 10.1186/s12890-015-0056-5 (PMC4446830; doi:10.1186/s12890-015-0056-5)
Supplement: Additional file 2: Table S2. — CFTR mutations found in individuals enrolled in the study. Gene and protein localization. Mutation classification and frequency from the present study are designated. Traditional and Human Genome Variation Society standard nomenclature for CFTR mutations are also indicated. [file 12890_2015_56_MOESM2_ESM.docx]

| **Supplement 2.** *CFTR* mutations found in individuals enrolled in the study. Gene and protein localization. Mutation classification and frequency from the present study are designated. Traditional and Human Genome Variation Society (HGVS) standard nomenclature^a^ for *CFTR* mutations are also indicated. | | | | | | | |
| --- | --- | --- | --- | --- | --- | --- | --- |
| Traditional nomenclature | HGVS nomenclature^a^ | | Localization (*CFTR* gene)^b^ | Consequence | Protein localization | Mutation classification | Predicted functional class |
|  | cDNA name | Protein name |  |  |  |  |  |
| F508del | c.1521_1523delCTT | p.Phe508del | Exon 10 | Point deletion | NBD1 | A | II |
| G542X | c.1624G>T | p.Gly542X | Exon 11 | Nonsense | NBD1 | A | I |
| R1162X | c.3484C>T | p.Arg1162X | Exon 19 | Nonsense | ICL4 | A | I |
| 3120+1G>A | c.2988+1G>A | - | IVS16 | Splicing | - | A | I |
| 1717-1G>A | c.1585-1G>A | - | IVS11 | Splicing | - | A | I |
| 1812-1G>A | c.1680-1G>A | - | IVS12 | Splicing | - | A | I |
| 2183AA>G | c.2051_2052delAAinsG | p.Lys684SerfsX38 | Exon 13 | Frameshift | RD | A | I |
| N1303K | c.3909C>G | p.Asn1303Lys | Exon 21 | Missense | NBD2 | A | II |
| R1066C | c.3196C>T | p.Arg1066Cys | Exon 17b | Missense | ICL4 | A | II |
| R553X | c.1657C>T | p.Arg553X | Exon 11 | Nonsense | NBD1 | A | I |
| I507V | c.1519A>G | p.Ile507Val | Exon 11 | Missense | NBD1 | A | - |

A = CF-causing mutation, B = CFTR-RD mutation, C = Mutation with no clinical consequence.

^a^Reference CFTR sequence accession number: NM_000492.3, nucleotide number 1 corresponds to the A of the ATG translation initiation codon; the reference sequence is numbered as 133.

^b^Traditional nomenclature.
