# Supplementary material for: Ventilatory abnormalities in patients with cystic fibrosis undergoing the submaximal treadmill exercise test
Source: BMC Pulm Med. 2015 May 19;15:63. doi: 10.1186/s12890-015-0056-5 (PMC4446830; doi:10.1186/s12890-015-0056-5)
Supplement: Additional file 3: Table S3. — Volumetric capnograph markers taking into account groups of patients with cystic fibrosis and healthy subjects. Time points analyzed by groups. [file 12890_2015_56_MOESM3_ESM.docx]

| **Supplement 3.** Volumetric capnograph markers taking into account groups of patients with cystic fibrosis and healthy subjects. Time points analyzed by groups. | | | | | | | | |
| --- | --- | --- | --- | --- | --- | --- | --- | --- |
| **Capnograph** | **Group*** | **Data** | **Time point** | | | | | **p-value^#^** |
|  |  |  | **1** | **2** | **3** | **4** | **5** |  |
| HR (bpm) | CF | Median ± SD | 90.33 ± 15.88 | 106.35 ± 15.17 | 110.16 ± 14.66 | 113.34 ± 14.91 | 94.32 ± 14.59 | **0.001** |
|  |  | Median | 87 | 103.5 | 108 | 112 | 93 |  |
|  | Control | Median ± SD | 89.35 ± 12.13 | 103.78 ± 11.07 | 107.64 ± 14.79 | 110.92 ± 11.73 | 97.50 ± 12.49 | **0.025** |
|  |  | Median | 89 | 101 | 108 | 108.5 | 98 |  |
|  |  | p-value | 0.819 | 0.819 | 0.635 | 0.250 | 0.066 |  |
| RR (rpm) | CF | Median ± SD | 21.83 ± 5.36 | 26.30 ± 8.79 | 26.71 ± 9.60 | 27.57 ± 9.46 | 24.11 ± 13.34 | **≤0.001** |
|  |  | Median | 22 | 25 | 24 | 27.5 | 22 |  |
|  | Control | Median ± SD | 20.63 ± 5.79 | 21.57 ± 5.94 | 22.80 ± 6.21 | 22.62 ± 6.24 | 20.09 ± 5.49 | **≤0.001** |
|  |  | Median | 20.50 | 22 | 23 | 23 | 20 |  |
|  |  | p-value | 0.301 | **0.018** | **0.049** | **0.002** | **0.038** |  |
| SpO_2_ (%) | CF | Median ± SD | 95.79 ± 3.46 | 94.81 ± 3.11 | 95.30 ± 2.26 | 94.92 ± 2.59 | 96.44 ± 1.79 | **≤0.001** |
|  |  | Median | 97 | 96 | 96 | 95 | 97 |  |
|  | Control | Median ± SD | 96.80 ± 2.83 | 96.57 ± 1.23 | 96.75 ± 0.70 | 96.09 ± 2.00 | 96.52 ± 1.92 | **≤0.001** |
|  |  | Median | 97 | 97 | 97 | 97 | 97 |  |
|  |  | p-value | **≤0.001** | **≤0.001** | **0.002** | **0.014** | 0.998 |  |
| EV (L/min) | CF | Median ± SD | 7.23 ± 1.94 | 10.90 ± 2.19 | 12.27 ± 2.82 | 13.53 ± 3.63 | 8.43 ± 2.46 | **≤0.001** |
|  |  | Median | 7.05 | 10.78 | 11.36 | 12.53 | 7.84 |  |
|  | Control | Median ± SD | 7.60 ± 2.26 | 10.78 ± 2.60 | 12.60 ± 3.63 | 13.38 ± 3.92 | 9.25 ± 3.60 | **≤0.001** |
|  |  | Median | 7.58 | 10.94 | 12.82 | 13.28 | 9.33 |  |
|  |  | p-value | 0.317 | 0.432 | 0.199 | 0.782 | **0.035** |  |
| VCO_2_ (mL/min) | CF | Median ± SD | 169.16 ± 57.26 | 281.57 ± 93.76 | 341.67 ± 120.66 | 384.37 ± 155.51 | 220.43 ± 88.31 | **≤0.001** |
|  |  | Median | 156.49 | 261.09 | 338.60 | 363.62 | 201.26 |  |
|  | Control | Median ± SD | 196.99 ± 59.52 | 322.24 ± 93.34 | 405.42 ± 141.58 | 451.00 ± 162.43 | 290.91 ± 128.54 | **≤0.001** |
|  |  | Median | 185.95 | 317.25 | 399.94 | 441.69 | 299.15 |  |
|  |  | p-value | **0.03** | **0.005** | **0.001** | **0.002** | **≤0.001** |  |
| Index (MV/VCO_2_) | CF | Median ± SD | 43.98 ± 7.53 | 41.42 ± 9.94 | 38.47 ± 9.20 | 37.85 ± 8.68 | 40.81 ± 7.81 | **≤0.001** |
|  |  | Median | 43 | 39.50 | 36 | 36 | 39.27 |  |
|  | Control | Median ± SD | 39.31 ± 6.91 | 34.78 ± 5.83 | 32.36 ± 4.67 | 30.75 ± 4.35 | 34.40 ± 8.48 | **≤0.001** |
|  |  | Median | 37.50 | 34 | 32 | 30.50 | 32 |  |
|  |  | p-value | **≤0.001** | **≤0.001** | **≤0.001** | **≤0.001** | **≤0.001** |  |
| PETCO_2_ (mmHg) | CF | Median ± SD | 35.82 ± 16.03 | 37.60 ± 5.37 | 38.76 ± 5.06 | 39.52 ± 4.83 | 34.61 ± 3.45 | **≤0.001** |
|  |  | Median | 34.14 | 38 | 38.82 | 39.95 | 34.88 |  |
|  | Control | Median ± SD | 36.07 ± 4.03 | 41.01 ± 3.63 | 42.12 ± 3.90 | 42.58 ± 4.28 | 37.13 ± 3.83 | **≤0.001** |
|  |  | Median | 37.25 | 41.84 | 42.53 | 42.30 | 37.13 |  |
|  |  | p-value | **0.002** | **≤0.001** | **≤0.001** | **≤0.001** | **≤0.001** |  |
| DS/TV | CF | Median ± SD | 0.26 ± 0.13 | 0.21 ± 0.06 | 0.19 ± 0.06 | 0.19 ± 0.06 | 0.22 ± 0.06 | **≤0.001** |
|  |  | Median | 0.24 | 0.21 | 0.19 | 0.18 | 0.22 |  |
|  | Control | Median ± SD | 0.25 ± 0.06 | 0.21 ± 0.05 | 0.20 ± 0.05 | 0.19 ± 0.06 | 0.22 ± 0.05 | **≤0.001** |
|  |  | Median | 0.25 | 0.20 | 0.19 | 0.18 | 0.21 |  |
|  |  | p-value | 0.424 | 0.918 | 0.484 | 0.905 | 0.989 |  |

SpO_2_, transcutaneous oxygen saturation; bpm, beats per minute; rpm, breaths per minute; EV, expiratory volume; VCO_2_, fraction of expired CO_2_; PETCO_2_, partial pressure of carbon dioxide in the air expired at the end of complete exhalation; L/min, liters per minute; mL/min, milliliters per minute; mmHg, millimeters of mercury; DS/TV, ratio between dead space and tidal volume; RR, respiratory rate; HR, heart rate; FEV_1_/FVC, ratio between forced expiratory volume in the first second of forced vital capacity. Data are shown as mean ± standard deviation; median. *The difference between the groups was evaluated by the Mann–Whitney *U* test (data not normally distributed). ^#^The difference between time points was calculated by the Friedman test. For all analyses, α = 0.05 was considered. Positive p-values are shown in bold.
