# Supplementary figures and images for: Ventilatory abnormalities in patients with cystic fibrosis undergoing the submaximal treadmill exercise test
Source: BMC Pulm Med. 2015 May 19;15:63. doi: 10.1186/s12890-015-0056-5 (PMC4446830; doi:10.1186/s12890-015-0056-5)

## Slide 1
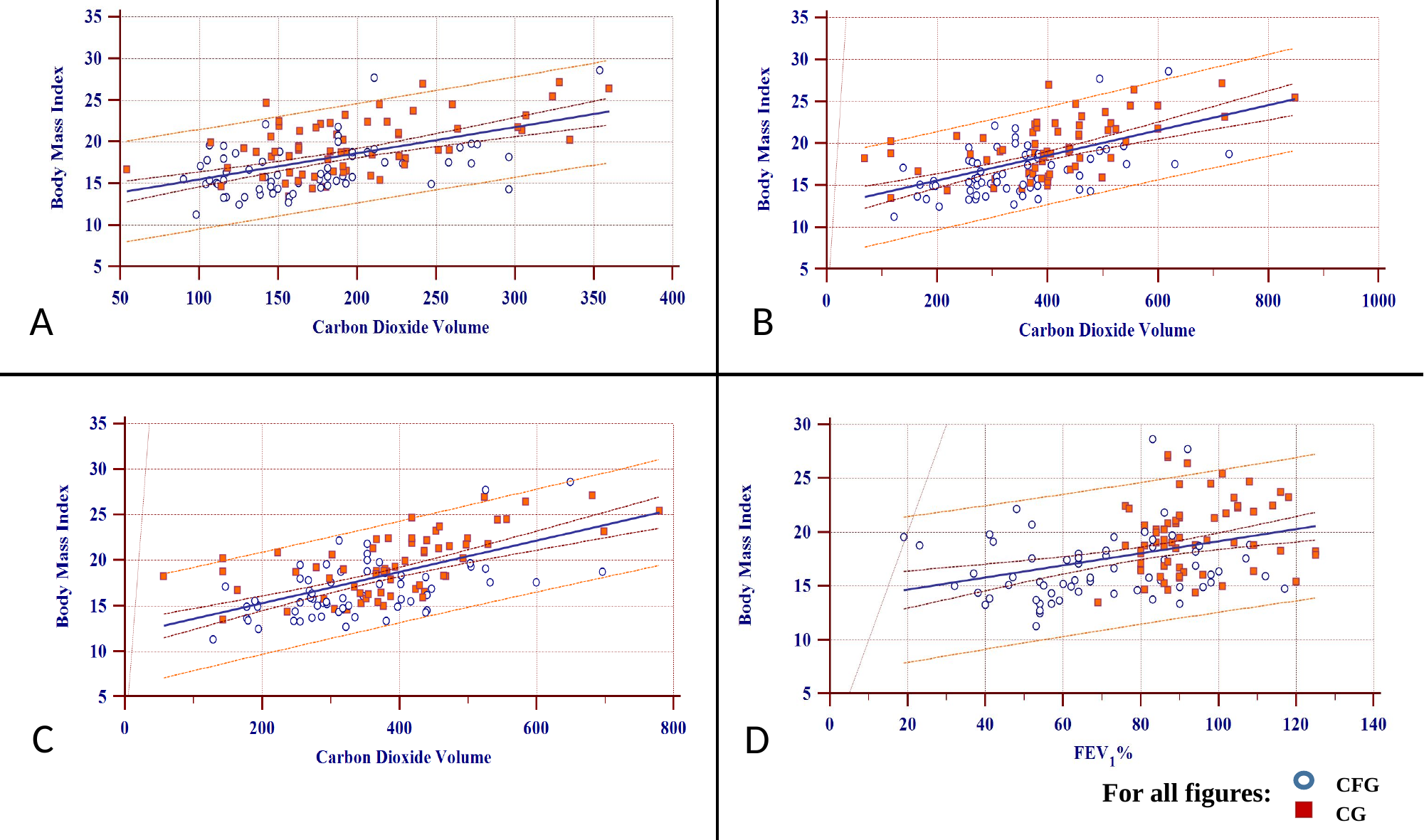

A
B
C
D
CFG
CG
For all figures:

Supplement: Additional file 4: Figure S4. — Linear regression of VCO2 and FEV1% by body mass index (BMI). A. Time point 1 - VCO2 by BMI - [Combined: y = 12.382 + 0.031 x (p ≤ 0.001)], [CF: y = 12.381 + 0.025 x (p = 0.001)], [Control: y = 13.810 + 0.029 x (p ≤ 0.001)]. B. Time point 3 - VCO2 by BMI - [Combined: y = 12.573 x + 0.015 (p ≤ 0.001)], [CF: y = 12.278 + 0.013 x (p < 0.001)], [Control: y = 14.244 + 0.013 x (p ≤ 0.001)]. C. Time point 5 - VCO2 by BMI - [Combined: y = 11.854 x + 0.017 (p ≤ 0.001)], [CF: y = 11.712 + 0.015 x (p ≤ 0.001)], [Control: y = 13.266 + 0.016 x (p ≤ 0.001)]. D. Time point 1 - FEV1% by BMI - [Combined: y = 13.566 + 0.056 x (p = 0.001)], [CF: y = 15.127 + 0.023 x (p = 0.196)], [Control: y = 14.919 + 0.049 x (p = 0.133)]. [file 12890_2015_56_MOESM4_ESM.pptx]
